# Understanding policy commitments for universal health coverage: a framework for analysis

**DOI:** 10.1186/s12961-025-01370-4

**Published:** 2025-07-16

**Authors:** Andréa Williams, Jesper Sundewall

**Affiliations:** 1 Division of Social Medicine and Global Health, Dept of Clinical Sciences, Lund University, Jan Waldenstroms Gata 35, 22100 Malmo, Sweden; 2 Health Economics and AIDS Research Division (HEARD), College of Law and Management, University of Kwa-Zulu Natal, University Road, 4000 Durban, South Africa; 3 Division of Clinical Epidemiology, Department of Clinical Research, University Hospital Basel, University of Basel, 4001 Basel, Switzerland

**Keywords:** Health policy, Framework, Developing countries, Policy analysis, Universal health coverage

## Abstract

**Background:**

Countries around the world have committed to universal health coverage (UHC), a global vision that affirms the right for all people to access essential healthcare, when and where they need it and regardless of their ability to pay. UHC, as a political commitment, developed as part of the Sustainable Development Agenda in 2015 and, more recently, at the United Nations High-Level meeting on UHC in 2019. A policy commitment to UHC means translating the broad vision of UHC into nationally appropriate, locally relevant health policies. The aim of this work is to develop an analytical framework for describing the key features of UHC to assess how UHC is conceptualised and translated at the national health policy level.

**Methods:**

We analysed purposively collected documents on UHC and conducted case studies of relevant health policies in three countries: South Africa, Botswana, and Kenya.

**Results:**

We propose a framework that includes five components we consider central to a UHC approach, namely: population coverage, healthcare service provision, health financing, health equity, and leadership and governance. The framework was applied to health policies in three countries in Africa (Botswana, Kenya, and South Africa) to test its relevance and applicability.

**Conclusions:**

Analysing policy commitments for UHC is central to understanding how countries are translating the broad aspiration into action. Our framework provides a useful tool by breaking down UHC into five core components and proposes questions to guide how policy commitments can be identified.

## Introduction

Today, many countries across the world are committed to moving towards universal health coverage (UHC); a global commitment that affirms the right of all people to receive healthcare on equal terms, regardless of their ability to pay. The concept of UHC evolved as a part of the broader Sustainable Development agenda in response to the need for wider, more equitable healthcare coverage across the globe [1]. Countries have committed to UHC through the 2030 Agenda (United Nations 2015) and, more recently, at the high-level meetings on UHC in 2019 and 2023 [2]. A commitment to UHC is largely aspirational and outlines how health services should be delivered and prioritized. UHC can therefore be understood as a shared set of values that guide policies and initiate action towards a clear goal, thereby making UHC a political matter rather than a technical one.

The path towards UHC is expected to look different in each country and will depend on a number of factors, including health system organisation, resources available, and political structures and governance [3]. As drivers of the UHC agenda, national governments are ultimately responsible for developing and implementing health policies that are aligned with UHC’s socioeconomic purpose. This means translating the broad principles of UHC into locally relevant and nationally appropriate health policies. Attention has been dedicated to discussing the definition of UHC, defining the services that should be prioritized and describing the mechanisms and extent to which services are delivered. However, less effort has been given to understanding how governments are translating principles of UHC into policies at the country level.

UHC within health policies can be understood as the articulation of key values that align with the broad UHC vision. There are several ways in which UHC is understood and articulated within national health policies, and how countries plan to achieve UHC will undoubtedly look different. However, UHC enshrines a few fundamental principles that every country will need to address through both policy and action. We therefore propose a framework that identifies key components to better understand how countries translate and articulate UHC by addressing core principles of UHC within national health policies. The framework was developed with the aim of supporting the assessment of health policies in selected countries where UHC is challenged by political, economic and structural factors. The development of the framework was guided by existing definitions of UHC and was informed by several key publications, including the World Bank’s “Universal Health Coverage in Africa: A Framework for Action” and the *2030 Global Agenda for Sustainable Development* [4].

## Methods

**Fig. 1:**
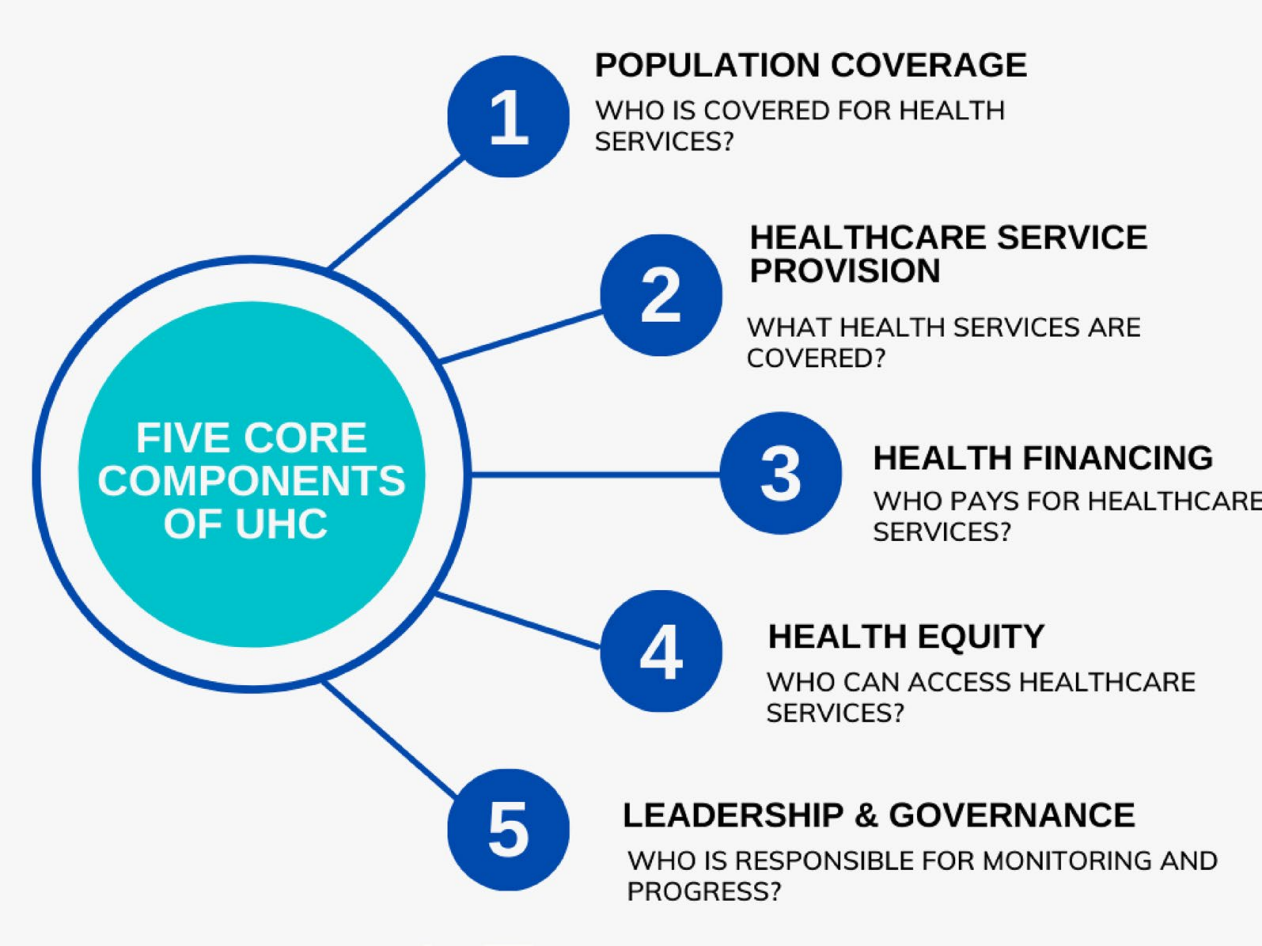
Five core components of UHC

Developing the core components required collating existing definitions of UHC from key reports and policy documents. We also purposively sampled and selected case countries to test the framework for its relevance and applicability. Our two-part framework proposes five core components that are central to UHC (Fig. 1) with a detailed table of key questions to guide policy analysis. Figure 1 shows five core components of UHC, which include population coverage, service provision, financing, equity, and leadership and governance. Each component is framed by a principal question that can be used to guide a broad analysis of policy content.

Table 1 provides a structure for a more detailed analysis, including several sub-questions under each component to help extract specific information from health policy documents. We have applied the framework to an analysis of UHC policies in three countries in Africa (Botswana, Kenya, and South Africa).

**Table 1:** Framework for analysing components of UHC in national health policies

Universal health coverage (UHC)				
Purpose of UHC	UHC asserts good health and well-being as the cornerstone of social and economic development. UHC aims to ensure all people have the right to access essential health services according to need while protecting them from financial risk [1]			
Core components of UHC and how these are articulated in health policy:				
1. Population coverage	2. Healthcare service provision	3. Health financing	4. Health equity	5. Leadership and governance in healthcare
Who is covered?	What health services are covered?	Who pays for health services?	Who can access health services?	Who is responsible for UHC monitoring and progress?
1.1 How does the policy describe the population who is eligible to receive healthcare? 1.2 How are vulnerable groups identified and described in the policy?	2.1 What healthcare services are considered to be high priority? 2.2 How are priority health services assigned? 2.3 What are the mechanisms for providing health services to the population? (i.e. integrating services into packages, gatekeeping, etc.)	3.1 How does the policy describe health system funding sources? (domestic, private and/or external) 3.2 How does the policy describe the purchasing-payment mechanism for health services? 3.3 How does the policy describe mechanisms for ensuring financial risk protection of individuals?	4.1 How is healthcare access described? (i.e. physical distance, population demand, healthcare capacity) 4.2 Does the policy include strategic interventions for the population who is unable to pay for health services at the point of access? 4.3 How does the policy describe the health system organisation by point of access to healthcare services?	5.1 How does the policy describe key stakeholders and their role in the oversight of UHC policy development and implementation? 5.2 Does the policy include the establishment of advisories, committees or teams to accelerate UHC progress? 5.3 Is there a transparent monitoring and evaluation framework in place to monitor UHC progress?

## Results

### Population coverage

Population coverage under UHC refers to the proportion of the population covered for a specific set of health services [5]. The aspiration for full population coverage under UHC is clear: all members of society should be able to access essential healthcare. How policies describe population coverage reflects their inclusivity. It is especially important that policies recognize and include socially vulnerable groups and those who require additional support in accessing healthcare. Applying the framework to Botswana’s national health policy provides an example of how population coverage has been described for the proportion of the population living with human immunodeficiency virus/acquired immune deficiency syndrome (HIV/AIDS). Population coverage is clearly articulated and focused on expanding coverage for HIV/AIDS treatment. This approach has led to Botswana achieving universal access for antiretroviral treatment and has prevented mother-to-child transmission for all women living with HIV/AIDS [6] (Box 1). Botswana is the first country in Africa to have achieved this milestone.

Box 1: Botswana’s universal coverage for antiretroviral treatment for persons living with HIV/AIDS Based on question 1.1 of the framework:

How does the policy describe the population eligible for healthcare?

Excerpt from Botswana’s National Policy on HIV and AIDS [7]:

“Access to appropriate [HIV] prevention methods will be equally ensured to all citizens of Botswana, without distinction by, but not limited to, ethnicity, gender, and age, religious or political affiliations” [7] (p. 12) […] “HIV testing services shall be available, on a non-discriminatory basis, to all citizens of Botswana” [7] (p. 13) […] “All HIV testing in the country is to be accompanied by referrals to appropriate services for prevention, care, treatment and support”. [7] (p. 14)

The number of new HIV/AIDS infections in Africa decreased by over a third in the past two decades [8]. Despite this progress, the impact of HIV/AIDS is still a major burden on Africa’s health systems. Similar to many countries in Africa, Botswana has prioritized infectious disease interventions on the national health agenda and has invested substantial resources in addressing the disease burden attributed to HIV/AIDS [9]. Botswana’s National Policy on HIV and AIDS is guided by national development guidelines and supported by global development partners. Botswana has become the first high-burden country to have successfully achieved universal coverage of anti-retroviral treatment and to eliminate mother-to-child transmission among people living with HIV/AIDS. Botswana is the first country in Africa to have successfully achieved this target.

### Healthcare service provision

Service provision under UHC refers to the inclusion and distribution of a defined set of healthcare services available to the population [1]. Delivering safe, quality and effective healthcare is an essential part of expanding UHC. The ambition for service provision under UHC is the inclusion of as many essential health services as possible. Our framework assesses the services that are covered under the policy, how these services have been identified and prioritized, and what mechanisms have been chosen for providing these healthcare services. The inclusion of services under a country’s health policy will largely depend on the its health profile. The services provided should reflect the burden of disease. Understanding the policy commitments for service provision can offer insight into health system efficiency and performance.

### Health financing

Sufficient and sustainable financing is critical for progressing towards UHC. Governments in low- and middle-income countries face economic constraints that limit their ability to adequately fund health systems [10]. This results in a reliance on household spending to supplement health financing, leading to a high burden of out-of-pocket healthcare expenses [11]. Our framework considers three key policy areas in health financing for UHC: funding sources for the health system, payment and purchasing of health services, and financial risk protection. The framework assesses how countries budget for and reduce the financial barriers to healthcare access through mechanisms such as prepayment or resource pooling to improve financial risk protection for the population. We applied the framework to South Africa’s National Health Insurance (NHI) policy which focuses on establishing a pooled fund for strategic purchasing of health services on behalf of the entire South African population [12] (Box 2). This shift in health financing will have implications for healthcare in South Africa, especially for addressing persistent inequities in health financing.

Box 2: South Africa’s National Health Insurance for financial risk protection Based on question 3.2 of the framework: How does the policy describe the purchasing-payment mechanism for health services?

Excerpt from policy National Health Insurance for South Africa: Towards Universal Health Coverage [12]:

“NHI will be established as a single-payer and single-purchaser fund responsible for the pooling of funds and the purchasing of personal health services on behalf of the entire population. The NHI Fund will be appropriately financed in order to be able to actively purchase personal health services for all who are entitled to benefit”. [12] (p. 49)

The public debate on healthcare in South Africa has been contentious for many years. South Africa is known to have high levels of social inequality that have permeated all spheres of society. South Africa’s health system is highly fragmented. The state-funded public sector serves approximately 70% of the total population, while the private sector, which is primarily funded through individual contributions, serves less than 30% [13]. The unequal distribution of finances has led to a discrepancy in the quality of healthcare services provided between the public and private sectors. To redress the balance, the South African government developed National Health Insurance (NHI) in 2012 as a mechanism to reduce health inequities through a centralized model for health financing. The role of the NHI is to pool tax-generated funds and purchase healthcare services on behalf of the entire population. Healthcare services will be strategically purchased through a single purchasing entity for all South Africans, regardless of status or income. The NHI will be implemented to create a modern, universal health financing system to protect individuals from the financial burden of healthcare costs.

### Health equity

Equity is a key component in progressing towards UHC. UHC implies access to healthcare on the basis of need and regardless of the ability to pay. However, in many countries, the proportions of healthcare benefits and needs are inversely distributed [14]. Our framework aims to identify how UHC policies address issues of equity by examining how they describe access to healthcare, outline strategic interventions for reducing barriers to healthcare access and whether the health system is built around a primary healthcare (PHC) approach. Applying the framework to Kenya’s national health policy, we were able to identify maternal and child health as a healthcare service priority. Expanding these services would improve health equity for this population. On the basis of policy recommendations, Kenya has developed and expanded essential maternal and child health services through an integrated PHC approach (Box 3).

Box 3: Kenya’s “Mama Linda” scheme for improving health equity for maternal and child health service users Based on question 4.1: How is healthcare access described? (i.e. physical distance, population demand, healthcare capacity)

Excerpt from Kenya’s national health policy [15]:

“All persons shall have adequate physical access to health and related services, defined as ‘living at least 5 km from a health service provider where feasible, and having the ability to access the health service’”. [15] (p. 37)

Excerpt from Kenya’s National Reproductive Health Policy (Ministry of Health, Kenya 2022):

“The Mama Linda programme will address urgent gaps in reproductive health” […] [15] (p. 36)) “[The program will] require that each pregnancy be registered at the nearest accredited health facility at the earliest opportunity and be enrolled into the government free maternal, new-born and infant health scheme”.

Kenya’s maternal and infant mortality rates are among the highest in the world [16]. Some challenges to service provision include a lack of accessible birthing centres, financial constraints that limit the ability to pay for health services, and limited empowerment for women to make health-related decisions and seek necessary antenatal care [17]. Kenya has prioritized the expansion of primary healthcare to decrease the physical barriers to healthcare access. Since 2013, Kenya has made maternal and child health services available free at the point of use at all public healthcare facilities. Kenya’s Ministry of Health has since formalized this arrangement through the development of the “Mama Linda” program, a social insurance scheme under the country’s National Hospital Insurance Fund. Mama Linda provides packages of basic ante- and postnatal services that are accessible to all pregnant women, regardless of financial status, at their nearest healthcare facility. The Mama Linda initiative has seen a significant increase in women seeking and receiving essential antenatal services, contributing to improved outcomes for women and children [18].

### Leadership and governance

Influential leadership and strategic governance are required for moving towards UHC, as this requires both political will and implementing health system reforms. Our framework assesses how a country’s UHC policies describe goals and targets and whether they include relevant indicators to evaluate progress over time. Moving towards UHC is a process that requires political commitment. This includes the mobilization of key stakeholders, establishment of oversight teams, and a transparent monitoring and evaluation system to track UHC progress [19]. Technical teams can support the implementation and monitoring of policies by tracking key indicators and targets. Furthermore, the establishment of monitoring and evaluation frameworks serve as a mechanism for accountability and continuity in policy implementation.

## Discussion and conclusions

Moving towards UHC is a global commitment that begins at the country level. As such, the framework can be used as a starting point for identifying policy gaps, recognizing health system areas that require attention, and supporting both advocacy and the implementation of new policies. UHC is a direction rather than a destination, and understanding policy commitments helps guide countries along that path. The broad definition of UHC means that countries have sufficient room to interpret and implement the UHC vision in a way that is contextually relevant. Our ambition has been to develop a framework that was concise and practical, offering a tool to analyse how countries choose to articulate and translate UHC within their national health policies. The value of this framework lies in its ability to assess how a political commitment translates into actionable and strategic policies. As countries have committed to UHC in different ways, our framework allows for a more detailed understanding of the extent to which existing policies align with each country’s ambition to advance UHC. A primary challenge in developing the framework was defining the components of UHC. UHC is an exceptionally broad concept that is difficult to break down into clearly defined components. The framework is not intended to oversimplify the vision of UHC but to encompasses a range of principles that guide policy action towards achieving an ambitious global goal.

## Data Availability

Policies analysed for this article are available from the corresponding author on request.

## Abbreviations

NHI: National health insurance
PHC: Primary healthcare
UHC: Universal health coverage
